# Efficacy and Safety of Transarterial Chemoembolization in Elderly Patients of Advanced Hepatocellular Carcinoma With Portal Vein Tumor Thrombus: A Retrospective Study

**DOI:** 10.3389/fonc.2021.646410

**Published:** 2021-07-07

**Authors:** Qinghe Tang, Wei Huang, Jun Liang, Junli Xue

**Affiliations:** ^1^ Department of Hepatobiliary and Pancreatic Surgery Department, Shanghai East Hospital, School of Medicine, Tongji University, Shanghai, China; ^2^ Department of Oncology, Shanghai East Hospital, School of Medicine, Tongji University, Shanghai, China; ^3^ Department of Intervention Therapy, Shanghai East Hospital, School of Medicine, Tongji University, Shanghai, China

**Keywords:** TACE, elderly patients, advanced HCC, PVTT, adverse event

## Abstract

**Objective:**

The aim of the current study was to evaluate the safety and efficacy of transcatheter arterial chemoembolization (TACE) in elderly patients diagnosed as advanced hepatocellular carcinoma (HCC) accompanied with different types of portal vein tumor thrombosis (PVTT).

**Methods:**

Elderly HCC patients aged 70-year-old and above from January 2015 to December 2019 were included in this retrospective study. Efficacy data including OS, PFS, DCR, and ORR and safety data were collected in the indicated groups. Outcomes of HCC patients in the TACE group were compared with those patients in the best supportive care (BSC) group. Subgroup analyses were also conducted in the patients with different types of PVTT.

**Results:**

Among 245 elderly HCC patients, 124 were enrolled in this study. Out of these, 50.0% (n=62) underwent BSC treatment while 50.0% (n=62) underwent TACE. There were no major differences in the baseline characteristics of the two treatment groups. TACE treatment was associated with better median OS compared with BSC alone (11.30 m *vs.* 7.80 m; *P*<0.001). Subgroup analyses showed that patients with type I and type II PVTT could benefit from TACE compared with BSC, based on that OS was 14.30 m *vs.* 7.80 m (*P*=0.007) and 13.00 m *vs.* 8.00 m (*P*=0.002), respectively. The DCR in the TACE group was 62.90%, and 17.74% in the BSC group (p<0.001). The proportion of ORR in TACE group was 35.48%, while 0.00% in the BSC group (p<0.001). Multivariable analyses showed that patients undergoing TACE treatment had 52% lower odds of mortality compared with patients undergoing BSC treatment (HR: 0.48; 95%CI: 0.32-0.72). Similarly, the media PFS was improved following TACE treatment (7.50 m *vs.* 4.00 m; *P*<0.001). TACE could significantly prolong the PFS in both type I and type II PVTT subgroups, without greatly significant improvement in type III PVTT patients (4.50 m *vs.* 2.70 m; *P*=0.103). Type III PVTT patients in the TACE group had more AEs than type I and type II PVTT patients. According to multivariable analyses, PVTT types (type III *vs.* type I-II) (HR: 2.18; 95%CI: 1.29-3.70; *P*=0.004), tumor diameter (>5 cm *vs.* ≤5 cm) (HR: 1.94; 95%CI: 1.28-2.93; *P*=0.002), and treatment (TACE *vs.* BSC) (HR: 0.48; 95%CI: 0.32-0.72; *P*<0.001) were independent indicators of overall survival.

**Conclusions:**

In elderly advanced HCC patients with PVTT, palliative TACE treatment can be an accessible effective measure to improve the OS and PFS for both type I and type II PVTT patients.

## Introduction

Hepatocellular malignancy is one of the prominent causes of death worldwide. According to the data of GLOBOCAN statistics in 2018, the primary liver cancer ranks sixth among malignant tumors with worldwide new cases of 841,080, and the mortality ranks second with 781,631 liver cancer-related deaths (1). Primary liver cancer is the fourth most common malignancy and the second leading cause of cancer-related death in China. Of note, hepatocellular carcinoma (HCC) is responsible for 85-90% of primary liver cancer. In the past few decades, there has been a tremendous increment in elderly patients not only in China but throughout the world. As life expectancy increases, the management of elderly HCC patients has become a global problem. It is well recognized that the majority of HCC patients are often diagnosed at a late stage (stages B or C) according to the Barcelona Clinic Liver Cancer (BCLC) system and have fewer opportunities to accept radical treatments such as surgical resection, liver transplantation, or percutaneous ablation (2). Furthermore, elderly patients exhibit shorter life expectancy and more comorbidities compared with younger patients, and thereby, physicians are apt to follow more conservative treatment approaches for this population.

At present, transcatheter arterial chemoembolization (TACE) is recommended as an essential first-line palliative choice for the patients who are poor candidates for surgical resection. It has been widely applied for HCC patients with BCLC stage B, or diagnosed as multinodular asymptomatic tumors with an optimal liver function but no macroscopic vascular invasion (MVI) or extrahepatic metastasis. Of note, patients with MVI have a contraindication to TACE (2, 3). Portal vein tumor thrombosis (PVTT) as a common kind of MVI is an important indicator of poor prognosis in HCC patients (4), and occurs in 20-70% of HCC patients, with a low median survival time of only 2-4 months (5, 6). As a result, treatments in elderly patients with HCC and PVTT are limited. Since TACE has a theoretical risk of ischemic damage to normal liver parenchyma, it has long been considered to be contraindicated in HCC patients with PVTT, especially for elderly patients (7).

On the basis of the BCLC groupings, for the patients with advanced HCC and PVTT, the tyrosine kinase inhibitor Sorafenib is regarded as a standard pharmacological therapy (8–10). Similarly, the American Association for the Study of Liver Diseases (AASLD) and the European Association for the Study of the Liver (EASL) guidelines also recommend Sorafenib. However, they do not recommend TACE for Child-Pugh A or B patients with PVTT, irrespective of the degree of PVTT (11, 12). Of note, medications such as Sorafenib or Lenvatinib are too expensive to be affordable for patients residing in developing countries including China. Furthermore, the rate of tumor response to Sorafenib is modest with less than three months of survival prolongation compared with placebo (9, 10). Even today, TACE is still a meaningful treatment for unresectable HCC patients with PVTT in Asia (13). The Japan Society of Hepatology proposed TACE for HCC patients with PVTT in second-order branches that have a good hepatic function (Child-Pugh A or B) and lack extrahepatic spread (14). Chinese clinical practice guidelines for transarterial chemoembolization of HCC also recommend TACE for HCC patients with PVTT in the following situations: the main portal vein is not completely blocked, or the portal veins are fully blocked but have abundant compensatory collateral circulation or portal blood flow can be recanalized by stenting (15). To date, very few studies have reported the TACE treatment for elderly HCC patients with PVTT.

Moreover, the treatment of TACE for elderly patients remains controversial. For instance, Yau et al. reported that TACE improved both OS and PFS in elderly patients compared with non-elderly patients (16). On the other hand, Cohen et al. showed similar survival patterns between elderly patients and non-elderly patients (17). Overall, the importance to study the TACE treatment cannot be understated due to its efficacy and affordability among elderly HCC patients. To this point, the objective of the current study was to investigate the efficacy and safety of TACE therapy in elderly HCC patients with PVTT, as well as the prognostic difference among the patients with different PVTT types.

## Materials and Methods

### Study Design and Patients

Among elderly HCC patients, about 245 patients with PVTT were reviewed retrospectively from January 2015 to December 2019 in Shanghai East Hospital. Out of them, 124 patients were enrolled in the current study, including 62 receiving best supportive care (BSC) and 62 receiving the TACE treatment. This study was approved by the Ethics Committee of Shanghai East Hospital. Since all the patient identities were anonymized, the requirement for informed consent was waived.

In this study, the inclusion criteria of patients were: 1) 70 years old or older; 2) initially diagnosed as HCC with PVTT or recurrent HCC with PVTT following surgical resection, and could not tolerate surgical resection again or refused further surgery; 3) no history of treatments such as radiofrequency ablation, transplantation, I^125^ seed implantation, percutaneous ethanol injection, TACE, or radiotherapy; 4) no systemic treatment such as Sorafenib, Lenvatinib, or checkpoint inhibitors; 5) liver function is Child-Pugh A or B; 6) European Cooperative Oncology Group performance status (ECOG) is 0-2; 7) adequate hematologic and renal functions; and 8) could be evaluated by Response Evaluation Criteria in Solid Tumors (mRECIST) criteria. The exclusion criteria of patients were: 1) have extrahepatic metastasis; 2) complete main portal vein obstruction without collateral circulation; 3) other cancers; 4) combined with targeted therapy, chemotherapy, or immunotherapy; and 5) have contraindications to arteriography or TACE.

HCC was diagnosed based on pathologic findings and/or the American Association for the Study of Liver Diseases criteria (11). The modified mRECIST criteria was applied to evaluate the response to the therapy (18), clarified as complete response (CR), partial response (PR), stable disease (SD), or progressive disease (PD). The objective response rate (ORR) was defined as (CR+PR)/total cases×100%, and disease control rate (DCR) was defined as (CR+PR+SD)/total cases×100%. The primary endpoint was overall survival (OS), and the secondary endpoint included progress free survival (PFS) and safety. OS and PFS were determined from the time of the initial diagnosis or recurrence of HCC to disease progression or death. PVTT was stratified according to Cheng’s PVTT classification system: Type I: Tumor thrombi involving segmental branches of portal vein; Type II: Tumor thrombi involving right/left portal vein; and Type III: Tumor thrombi involving the main portal vein and trunk (19).

### TACE Procedure

The TACE procedure was performed as described previously (20). Overall, a selective 5 French catheter was utilized, and visceral angiography was performed out to evaluate the liver cancer feeding artery. The tip of the microcatheter was advanced into the hepatic segmental or tumor-feeding artery. An emulsion of 1-20 ml of lipiodol (Iodinated Oil Injection, Luyin pharmacy, China) and 40 mg of doxorubicin hydrochloride were administered into the feeding vessels. However, if the flow of the tumor feeding artery did not lower after injecting 20 ml, we continued to inject 150-1400 μm gelatin sponge to embolize the vessel, until we could observe a significantly slower rate of flow. The general treatment cycle of TACE was 4-6 weeks and then other TACE cycles were conducted out according to the results of the follow-up contrast-enhanced CT or MRI.

### Follow-Up

All patients were reevaluated one month after TACE. TACE would be repeated with an interval of 4-6 weeks if necessary. If the tumor had no viability based on contrast-enhanced CT or the patients had contradictions to TACE, the treatment of TACE would not be performed. The follow-up program included vital signs, serum AFP, liver function, coagulation function, chest CT scan, and abdominal contrast-enhanced CT or MRI scan every 6-8 weeks for the first year and every 3 months thereafter. Side effects of TACE were reported according to NCI-CTCAE version 5.0. All patients were followed up until death or until June 30, 2020.

### Statistical Analyses

Demographic characteristics and clinical outcomes were compared among patients undergoing TACE or BSC treatment. All categorical and continuous data were analyzed using descriptive statistics. Categorical variables were reported as total count and frequencies (%) while continuous variables were reported as median and interquartile ranges (IQR). Bivariate analyses were performed using the chi-squared test or Fisher exact test (2-tailed) for categorical variables, as appropriate, to assess the differences in the TACE and BSC treatment groups. Means for continuous variables that were normally distributed were compared using independent samples t-test. Survival curves were analyzed using the Kaplan-Meier method and compared using the log-rank test. The bivariate and multivariable Cox proportional hazard modeling was performed to identify independent prognostic factors based on adjusted hazard ratio and its associated 95% confidence interval (CI). Model selection was based on the stepwise technique to assess factors associated with overall survival. All statistical analyses were performed using IBM SPSS 23.0, and *P*<0.05 was considered statistically significant.

## Results

### Patient Demographics and Clinical Characteristics

**Figure 1:**
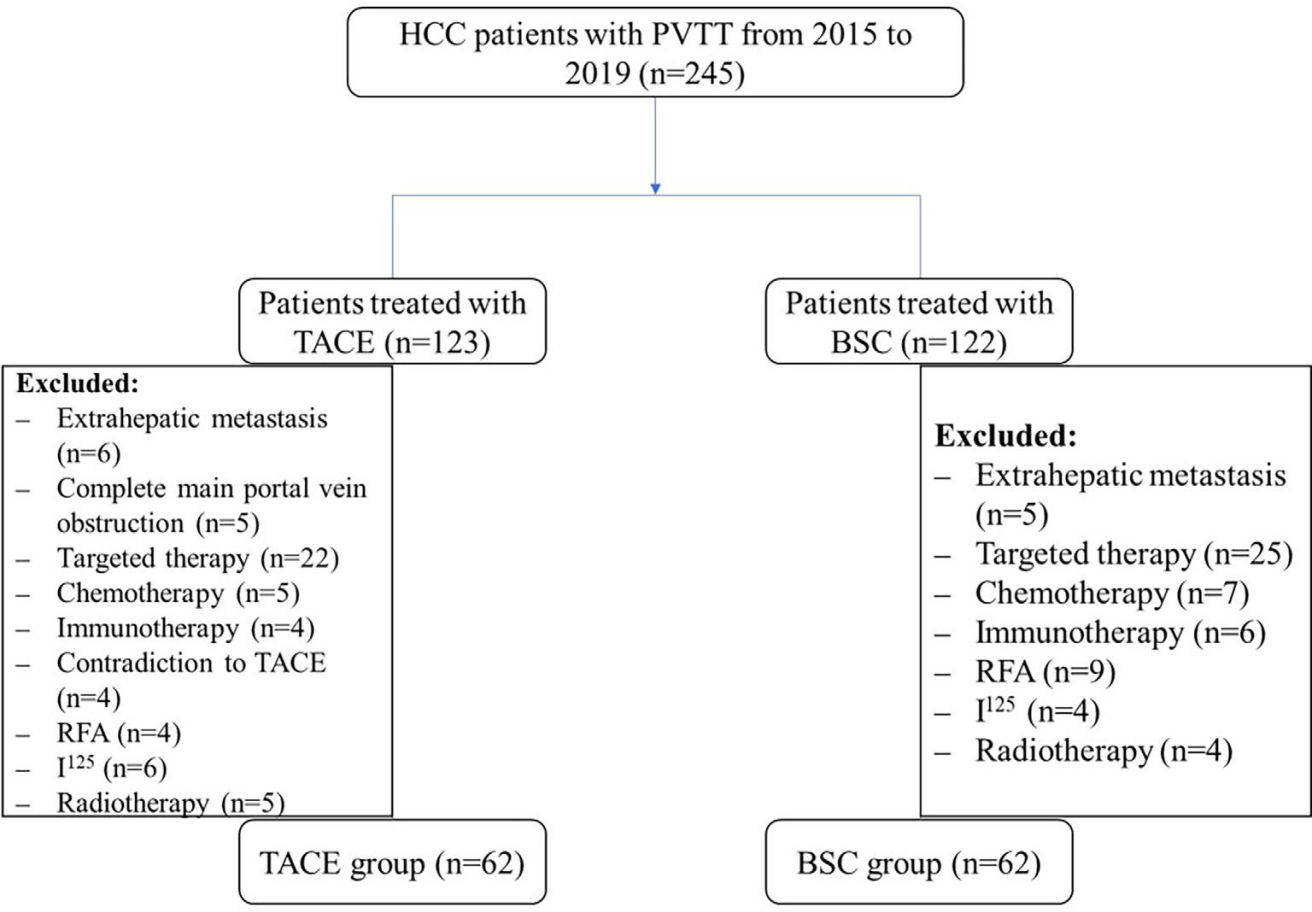
Flowchart of elderly HCC patients with PVTT recruitment.

Between January 2015 and December 2019, 245 elderly patients with unresectable HCC and PVTT undergoing treatment with TACE or BSC were collected, of which 124 were included in the final analysis (**Figure 1**). 50.0% (n=62) of the patients were treated with BSC, and 50.0% (n=62) was treated by the TACE therapy. Demographic characteristics were comparable among both the groups (*P*>0.05). Similarly, tumor size, tumor number, and total bilirubin were comparable among both the groups. The median levels of the ALT, AST, and D-Dimer among elderly HCC patients were higher in the BSC group compared with the TACE group (median BSC *vs.* TACE, ALT: 55.5 *vs.* 35.5, AST: 71.0 *vs.* 34.5, D-Dimer: 3.66 *vs.* 2.58; all *P*<0.05) (**Table 1**).

**Table 1:** Baseline characteristics of elderly HCC patients in the BSC group and the TACE group.

Variables	BSC group (n=62)	TACE group (n=62)	*P* value
**Gender**			
**Male n(%)**	51 (82.25)	53 (85.48)	0.63
**Female n(%)**	11 (17.75)	9 (14.52)	
Age*	75 (73, 78)	74 (72, 78)	0.33
**PVTT type**			
**Type I n(%)**	21 (33.87)	28 (45.16)	0.44
**Type II n(%)**	28 (45.16%)	23 (37.10)	
**Type III n(%)**	13 (20.97%)	11 (17.74)	
**Child-Pugh**			
**A n(%)**	58 (93.54)	59 (95.16)	0.70
**B n(%)**	4 (6.56)	3 ( 4.84)	
**ECOG**			
**0 n(%)**	11 (17.74)	18 ( 29.03)	0.20
**1 n(%)**	49 (79.03)	40 ( 64.52)	
**2 n(%)**	2 (3.23)	4 (6.45)	
Tumor size (cm)*	6.0 (4.0 - 7.0)	5.0 (4.0 – 7.0)	0.26
**Tumor number**			
**single n(%)**	4 (6.45)	3(4.84)	0.697
**multiple n(%)**	58 (93.55)	59(95.16)	
**Hepatitis**			
**Hepatitis B n(%)**	62 (100.00)	61(98.39)	0.32
**Hepatitis C n(%)**	0 (0.00)	1(1.61)	
**AFP**			
**≥400 n(%)**	17 (27.4%)	15 (24.2%)	0.68
**<400 n(%)**	45 (72.6%)	47 (75.8%)	
Total bilirubin*	14.5 (11.0, 20.0)	14.0 (10.0, 21.0)	0.89
Albumin*	38.5 (35.0, 40.0)	39.0 (36.0, 41.0)	0.28
ALT*	55.5 (34.0, 80.5)	35.5 (49.0, 21.0)	**<0.001**
AST*	71.0 (37.0, 85.5)	34.5 (23.0, 50.0)	**<0.001**
D-Dimer*	3.66 (2.09, 4.98)	2.58 (1.27, 3.88)	**0.039**

*data are median. p < 0.05 was considered statistically significant. Demographic characteristics were comparable among both the groups (p>0.05). The median levels of the ALT, AST, and D-Dimer among elderly HCC patients were higher in the BSC group compared with the TACE group (all p < 0.05).

ECOG, Eastern Cooperative Oncology Group; AFP, Alpha–fetoprotein; ALT, Alanine aminotransferase; AST, Aspartate aminotransferase. Bold values marked the significant P values (P < 0.05).

### Safety

The most common AEs related to TACE treatment observed in this study were fever (45.16%), liver dysfunction (41.94%), abdominal pain (35.48%), and anorexia (33.87%), and D-Dimer elevation (33.87%) (**Table 2**). Nausea, fatigue, ventosity, and diarrhea were also observed frequently. There is one upper GI hemorrhage (1.61%) after TACE treatment, which was under control after treatment. Subgroup analyses revealed that type III PVTT patients suffered more liver dysfunction, D-Dimer elevation, and ventosity than type I or type II PVTT patients (*P*<0.0167). Most of the AEs were grade 1/2 and well tolerated. The most common grade 3/4 AEs was fever and occurred in 10 (16.13%) patients. All these findings returned to the pre-treatment levels within less than one month after TACE.

**Table 2:** Adverse events related to the TACE treatment. Data are presented as number (%) of patients.

AE n(%)	All events							Grade 1-2	Grade 3-4
	Total (n=62)	PVTT I(n=28)	PVTT II(n=23)	PVTT III(n=11)	*P* value*				
					I *vs* II	I *vs* III	II *vs* III		
**Fever**	28 (45.16)	9 (32.14)	11 (47.83)	8 (72.73)	0.388	0.033	0.271	18 (29.03)	10 (16.13)
**Liver dysfunction**	26 (41.94)	8 (28.57)	8 (34.78)	10 (90.91)	**0.764**	**0.001**	**0.003**	19 (30.65)	7 (11.29)
**Abdominal pain**	22 (35.48)	8 (28.57)	8 (34.78)	6 (54.55)	0.764	0.068	0.151	22 (35.48)	0 (0.00)
**Anorexia**	21 (33.87)	8 (28.57)	6 (26.09)	7 (63.64)	1.000	0.068	0.060	21 (33.87)	0 (0.00)
**D-Dimer elevation**	21 (33.87)	5 (17.86)	6 (26.09)	10 (90.91)	**0.514**	**<0.001**	**0.001**	21 (33.87)	0 (0.00)
**Nausea**	17 (27.42)	7 (25.00)	5 (21.74)	5 (45.45)	1.000	0.262	0.232	17 (27.42)	0 (0.00)
**Fatigue**	17 (27.42)	7 (25.00)	6 (26.09)	4 (36.36)	1.000	0.694	0.692	16 (25.80)	1 (1.61)
**Ventosity**	14 (22.58)	3 (10.71)	5 (21.74)	6 (54.55)	**0.442**	**0.008**	**0.114**	14 (22.58)	0 (0.00)
**Diarrhea**	5 (8.06)	2 (7.14)	2 (8.70)	1 (9.09)	1.000	1.000	1.000	5 (8.06)	0 (0.00)
**Upper GI hemorrhage**	1 (1.61)	0	0	1 (9.09)	–	0.282	0.324	1 (1.61)	0 (0.00)

*p<0.0167 was considered statistically significant. Type III PVTT patients suffered more liver dysfunction, D-Dimer elevation, and ventosity than type I or type II patients.

### Efficacy

#### Overall Survival

**Figure 2:**
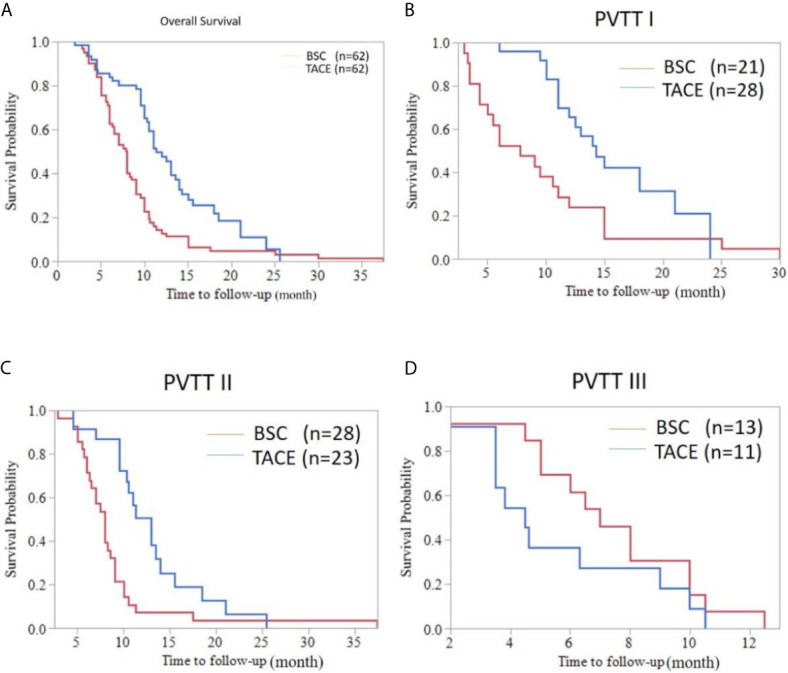
Overall survival curve of HCC patients with PVTT accepting the TACE or BSC treatment. **(A)** Whole population survival curve for the TACE group and the BSC group (median OS [months], 11.30 (9.636-12.964) *vs.* 7.80 (6.748-8.852); *P* < 0.001). **(B)** Survival curve of HCC patients with type I PVTT in the TACE group and the BSC group (OS, 14.30 (11.492-17.108) *vs.* 7.80 (3.875-11.725); *P*=0.007). **(C)** Survival curve of HCC patients with type II PVTT in the TACE group and the BSC group (OS, 13.00 (10.539-15.461) *vs.* 8.00 (6.987-9.103); *P*=0.002). **(D)** Survival curve of HCC patients with type III PVTT in the TACE group and the BSC group (OS, 4.50 (3.313-5.687) *vs.* 7.00 (5.239-8.761); *P*=0.176).

As shown in **Figure 2**, the median overall survival (OS) of elderly patients in the TACE and BSC groups was 11.30 months (95%CI: 9.636-12.964) and 7.80 months (95%CI: 6.748-8.852), respectively (*P*<0.001). In subgroup analyses, the median OS of type I PVTT patients was 14.30 months (95%CI: 11.492-17.108) and 7.80 months (95%CI: 3.875-11.725) in the TACE and BSC groups, respectively (*P*=0.007). Interestingly, in the type II PVTT group, the median OS of the TACE and BSC groups were 13.00 months (95%CI: 10.539-15.461) and 8.00 months (95%CI: 6.987-9.103), respectively (*P*=0.002). However, the OS of type III PVTT patients in the TACE group was poorer than the BSC group, which were 4.50 months (95%CI: 3.313-5.687) and 7.00 months (95%CI: 5.239-8.761), respectively (*P*=0.176).

#### Progress Free Survival

**Figure 3:**
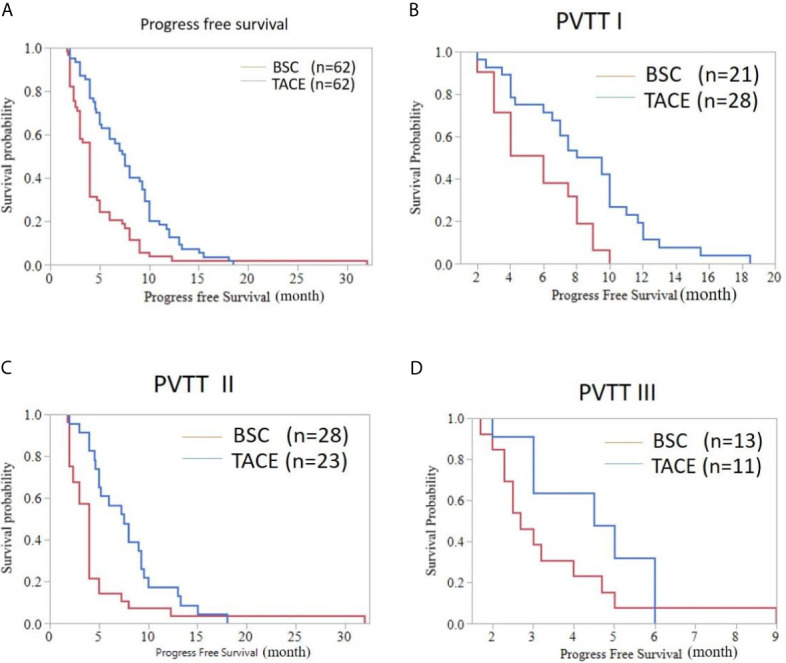
Progress free survival curve of HCC patients with PVTT accepting the TACE or BSC treatment. **(A)** Whole population (median PFS [months], 7.50 (6.284-8.716) *vs.* 4.00 (3.625-4.375); *P* < 0.001). **(B)** Patients with type I PVTT (PFS, 8.00 (5.485-10.515) *vs.* 6.00 (3.973-8.027); *P*=0.003). **(C)** Patients with type II PVTT (PFS, 7.50(5.152-9.848) *vs.* 4.00 (3.574-4.426); *P*=0.005). **(D)** Patients with type III PVTT (PFS, 4.50 (2.337-6.663) *vs.* 2.70 (2.083-3.317); *P*=0.103).

The median PFS was 4.00 months (95%CI: 3.625-4.375) for HCC patients with BSC treatment and was 7.50 months (95%CI: 6.284-8.716) for HCC patients treated with TACE (*P*<0.001). The TACE treatment prolonged the PFS in the three subgroups. In comparison of TACE and BSC, the PFS of type I PVTT patients were 8.00 months (95%CI: 5.485-10.515) and 6.00 months (95%CI: 3.973-8.027), respectively (*P*=0.003). For type II PVTT patients, the PFS in the TACE and BSC groups were respectively 7.50 months (95%CI: 5.152-9.848) and 4.00 months (95%CI: 3.574-4.426) (*P*=0.005). Type III PVTT patients could also benefit from TACE treatment. The PFS of type III PVTT patients in the TACE group was 4.50 months (95%CI: 2.337-6.663), while in the BSC group was 2.70 months (95%CI: 2.083-3.317), without great significance (*P*=0.103) (**Figure 3**).

#### Response Rate

**Figure 4:**
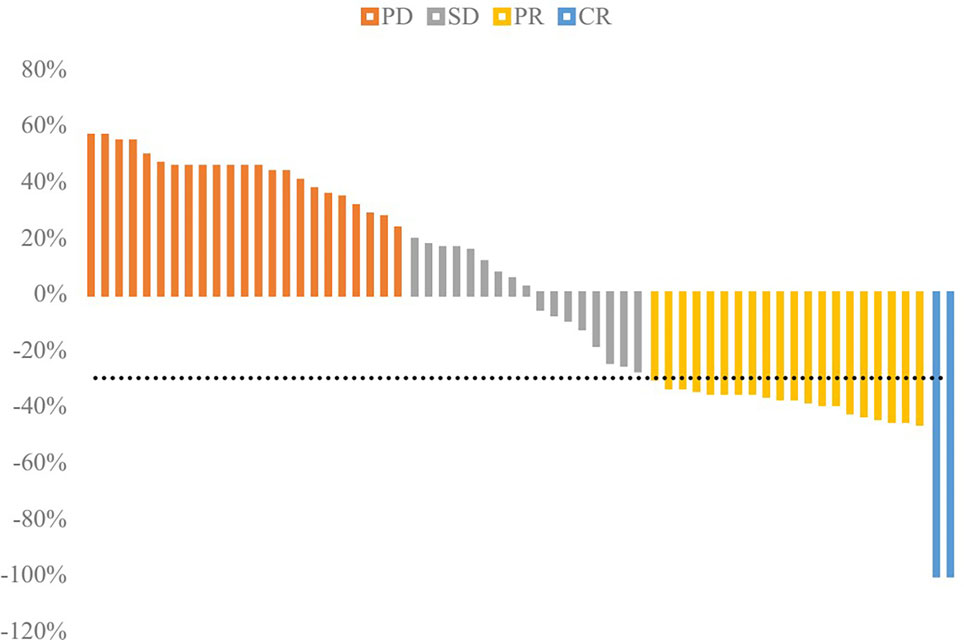
Waterfall plot of response rate in the TACE group. The disease control rate (DCR) was 62.90%, and objective response rate (ORR) was 35.48%.

The response rate was separately 35.48% in the TACE group and 0.00% in the BSC group and the response rate of TACE was significantly better (*P*<0.001). The DCR was separately62.90% in the TACE group and 17.74% in the BSC group (*P*<0.001). The proportions of CR, PR, SD, and PD were 3.22%, 32.26%, 27.42%, and 37.10% in the TACE group while 0.00%, 0.00%, 17.74%, and 82.26% in the BSC group. The waterfall plot figure was indicated in **Figure 4**, showing the response rate of HCC patients with PVTT in the TACE group.

#### Bivariate and Multivariable Cox Proportional Hazard Modeling Analyses

On bivariate analyses, the clinical factors such as PVTT types, ECOG performance status, tumor size, treatments (TACE *vs.* BSC), and tumor numbers were associated with the overall survival. On the multivariable regression analyses, it was found that the patients with type III PVTT had 118% higher hazards of mortality than those with type I-II PVTT (HR: 2.18; 95%CI: 1.29-3.70; *P* = 0.004). Interestingly, elderly HCC patients receiving the TACE treatment had 52% lower hazards of mortality than those receiving the BSC treatment (HR: 0.48; 95%CI: 0.32-0.72; *P* = 0.004). Based on these findings, multivariable Cox proportional hazards regression analyses revealed that PVTT types (type III *vs.* type I-II) (HR: 2.18; 95%CI: 1.29-3.70; *P*=0.004) and tumor diameter (>5 cm *vs.* ≤5 cm) (HR: 1.94; 95%CI: 1.28-2.93; *P*=0.002) were independent indicators of overall survival (**Table 3**).

**Table 3:** Bivariate and multivariable Cox proportional hazard modeling analyses for overall survival.

Variable	Bivariate analysis			Multivariate analysis		
	HR	CI	*P* value	HR	CI	*P* value
**Gender(F/M)**	1.12	0.68- 1.86	0.660			
**Age(<75/≥75)**	1.27	0.87- 1.85	0.218			
**Child-Pugh(B/A)**	1.36	0.63- 2.95	0.431			
**PVTT type(III/I-II)**	2.46	1.47- 4.09	**<0.001**	2.18	1.29-3.70	**0.004**
**ECOG(1-2/0)**	2.41	1.50- 3.86	**<0.001**			
**Tumor size(>5cm/≤5cm)**	1.91	1.29- 2.83	**0.0012**	1.94	1.28-2.93	**0.002**
**Albumin(≥35/<35g/L)**	0.98	0.62- 1.52	0.909			
**TACE *vs* BSC**	**0.61**	**0.42- 0.89**	**0.010**	0.48	0.32-0.72	**0.004**
**Total bilirubin(≥20/<20umol/L)**	1.59	1.06- 2.38	**0.024**			
**Hepatitis (C/B)**	0.72	0.10- 5.17	0.741			
**AFP(<400/≥400 ng/ml)**	0.81	0.53- 1.24	0.342			
**Tumor number(≥3/<3)**	2.37	1.03- 5.44	**0.042**			
**ALT(<40/≥40U/L)**	0.83	0.57- 1.23	0.359			
**AST(<60/≥60U/L)**	0.94	0.64- 1.39	0.759			
**D-Dimer (<0.55/≥0.55mg/L)**	1.06	0.62- 1.79	0.841			

Elderly HCC patients received the TACE treatment had 52% lower hazards of mortality compared with the BSC group patients (p<0.001). Multivariable Cox proportional hazards regression analysis revealed that PVTT types (type III vs type I-II) and tumor diameter (>5cm vs ≤ 5cm) (p<0.005) were independent indicators of overall survival.

ECOG, Eastern Cooperative Oncology Group; AFP, Alpha–fetoprotein;ALT, Alanine aminotransferase; AST, Aspartate aminotransferase; TACE, Transarterial Chemoembolization. Bold values marked the significant P values (P < 0.05).

## Discussion

In China, there is an increasing trend in the incidence of HCC patients. With the prolongation of life expectancy, treatment of elderly HCC patients has been recently a new challenge for global healthcare system. Due to the high prevalence of hepatitis B infection and low screening rate of early-stage liver cancer, most of HCC patients are usually diagnosed at an advanced stage. Among them, PVTT incidence can be as high as 70% (6). In addition, the patients suffering from advanced HCC with PVTT exhibit a poor prognosis, with median survival of only 2-4 months (21). Sorafenib is currently recommended as the standard first-line treatment by the American Association for the Study of Liver Diseases (AASLD) and the European Association for the Study of the Liver (EASL) for Child-Pugh A or B patients with PVTT (11, 12). However, the SHARP study revealed that the survival benefit of advanced HCC patients with Sorafenib administration was less than three months (9). In 2018, the randomized phase III non-inferiority trial REFLECT study revealed that the non-inferiority had achieved with a median OS of 13.6 months in the Lenvatinib group and 12.3 months in the Sorafenib group. Median PFS was 7.4 months and 3.7 months in Lenvatinib and Sorafenib groups, respectively. The study has met both the primary and secondary end points (22). Thereby, Europe, the Middle East, and Africa (EMEA), the American Food and Drug Administration (FDA), and National Medical Products Administration (NMPA) in China have approved Lenvatinib for the first-line treatment of both young and elderly unresectable HCC. Although Lenvatinib and Sorafenib could improve the survival of advanced HCC, these drugs have not yet been covered by the common medical insurance in most regions of China. The cost of Lenvatinib is as high as 48,000 yuan (nearly $7,000) per month, which limits its clinical application due to poor affordability. Compared to these drugs, TACE as an effective treatment measure with a low cost still plays an important role in advanced HCC patients. Although international guidelines do not recommend TACE for HCC patients with PVTT, TACE is still widely applied in clinical practice in Asia and recommended by both Chinese and Japanese guidelines (14, 15).

However, presently, there is no clear consensus on the efficacy of TACE for elderly HCC patients with PVTT. As such, we conducted this retrospective study to compare the survival in HCC patients treated with TACE *versus* BSC. The current study revealed that HCC patients above the age of 70 years old and receiving the TACE treatment had 52% times lower hazards of mortality compared with those treated with the BSC treatment. Subgroup analyses showed that the prognosis and response rate of the TACE treatment in HCC patients with different PVTT types were different. Compared with the BSC group, the TACE group significantly prolonged the OS and PFS of type I and type II PVTT patients. In contrast, among the type III PVTT patients, the results of PFS were comparable between the two groups. However, the OS of HCC patients with type III PVTT were even worse in the TACE group than that of the BSC group. The reason might be that the patients with type III PVTT were more likely to have TACE-related adverse effects and were at a later stage of the tumor, and thus they could not benefit from the TACE treatment.

Similar studies reported controversial results for the efficacy of the TACE treatment in HCC patients with type III PVTT. Liang et al. reported no survival benefit of TACE for the patients with the main portal vein thrombosis (20), while Yuan J et al. reported TACE benefit of type III PVTT patients (23). The discordances among the results of such clinical studies may be reduced to the selection bias among the subjects in different clinical studies, or the different regimens for the TACE treatment. These data suggested that large scale phase III clinical trials may be required to verify how TACE treatment could benefit advanced HCC patients with type III PVTT. Similar to our results, Chung et al. and Bai et al. proposed that HCC with type I/II PVTT could benefit from the TACE treatment in both young and elderly patients (24, 25). Based on the previous results and our research, the TACE treatment could be selected for elderly HCC patients with type I and type II PVTT if there are no contradictions, in order to find the chance of survival benefit.

The safety analysis showed that, although the adverse effects such as fever, abnormal liver function, and abdominal pain increased in the TACE group, most of them were grade 1 to 2 according to CTCAE 5.0 and could return to normal within one month. Subgroup analyses revealed that the incidences of adverse effects were higher in the type III PVTT group, especially liver dysfunction, D-Dimer elevation, and ventosity with great significance. It should be noted that D-Dimer obviously was upregulated following TACE treatment, especially in the type III PVTT group, which indicated possible hypercoagulation following the TACE treatment. Therefore, more attention should be paid to monitor the coagulation after surgery to avoid the possibility of embolism due to tumor-related or TACE-related hypercoagulation.

Bivariate analyses revealed that PVTT types, ECOG performances status, tumor size, tumor numbers, and treatment groups (TACE *vs.* BSC) were correlated with advanced HCC. On multivariable analyses, it was found that the type I and type II PVTT, tumor size (≤5 cm), and patients treated with TACE were advantageous independent indicators of HCC patients’ overall survival. Overall, the data suggested the necessity to fully evaluate the risks and benefits of HCC patients in the clinical practice, in order to make a suitable strategy to maximize the benefits of patients.

Currently, the combination strategies of TACE with other treatments, such as TACE combined with surgery, radiotherapy, seed implantation, Sorafenib administration, and other treatment modes are being discussed (23, 25–29). However, the subjects and results of these clinical studies varied greatly. Therefore, it is also necessary to explore the optimal combination strategies of TACE and other treatments, in order to maximize the benefits to the patients.

There were some limitations to the study. First, it is a retrospective study, and there may be a selection bias during the enrollment of subjects. Second, it is a single-center study with a small sample size which could not be fully representative, and thus large-scale phase III clinical trials or multicenter study are recommended to confirm these results. Third, the evaluation of clinical effects on basis of mRECIST criteria may be biased because of the investigator-independent factors. Independent Review Committee (IRC) is still in need of future studies to increase the generalizability.

In conclusion, the retrospective study showed that palliative TACE treatment could prolong the OS and PFS of elderly advanced HCC patients with type I and type II PVTT without severe adverse events. The elderly patients diagnosed as HCC had 52% lower hazards of mortality compared with those treated in the BSC group. For these patients who could not afford the standard first-line drugs such as Lenvatinib or Sorafenib, TACE is still an accessible effective measure.

## Data Availability Statement

The original contributions presented in the study are included in the article/supplementary material. Further inquiries can be directed to the corresponding authors.

## Ethics Statement

The studies involving human participants were reviewed and approved by Ethics Committee of Shanghai East Hospital. The ethics committee waived the requirement of written informed consent for participation.

## Author Contributions

JL and WH collected the clinical data. QT processed the statistical data. JX drafted the manuscript. JX and QT revised the final manuscript. All authors contributed to the article and approved the submitted version.

## Funding

This study was funded by the Outstanding Clinical Discipline Project of Shanghai Pudong (No.PWYgy2018-02; study design and data collection) and the Science and Technology Commission of Shanghai Municipality (Grant number: 19DZ1910502, to JX; data analysis and manuscript revision).

## Conflict of Interest

The authors declare that the research was conducted in the absence of any commercial or financial relationships that could be construed as a potential conflict of interest.
